# Three-dimensional printed hydroxyapatite bone tissue engineering scaffold with antibacterial and osteogenic ability

**DOI:** 10.1186/s13036-021-00273-6

**Published:** 2021-08-09

**Authors:** Liu Zhongxing, Wu Shaohong, Li Jinlong, Zhang Limin, Wang Yuanzheng, Gao Haipeng, Cao Jian

**Affiliations:** 1 Department of Orthopedics, Affiliated Hospital of Chifeng University, Inner Mongolia 024000 Chifeng, People’s Republic of China; 2 Department of Stomatology, Affiliated Hospital of Chifeng University, Inner Mongolia 024000 Chifeng, People’s Republic of China; 3 Department of Ophthalmology, Affiliated Hospital of Chifeng University, Inner Mongolia 024000 Chifeng, People’s Republic of China

**Keywords:** Bone scaffold, 3D printed, Hydroxyapatite, Osteoinduction, Antibacterial

## Abstract

The development of an effective scaffold for bone defect repair is an urgent clinical need. However, it is challenging to design a scaffold with efficient osteoinduction and antimicrobial activity for regeneration of bone defect. In this study, we successfully prepared a hydroxyapatite (HA) porous scaffold with a surface-specific binding of peptides during osteoinduction and antimicrobial activity using a three-dimensional (3D) printing technology. The HA binding domain (HABD) was introduced to the C-terminal of bone morphogenetic protein 2 mimetic peptide (BMP2-MP) and antimicrobial peptide of PSI10. The binding capability results showed that BMP2-MP and PSI10-containing HABD were firmly bound to the surface of HA scaffolds. After BMP2-MP and PSI10 were bound to the scaffold surface, no negative effect was observed on cell proliferation and adhesion. The gene expression and protein translation levels of type I collagen (COL-I), osteocalcin (OCN) and Runx2 have been significantly improved in the BMP2-MP/HABP group. The level of alkaline phosphatase significantly increased in the BMP2-MP/HABP group. The inhibition zone test against *Staphylococcus aureus* and *Escherichia coli* BL21 prove that the PSI10/HABP@HA scaffold has strong antibacterial ability than another group. These findings suggest that 3D-printed HA scaffolds with efficient osteoinduction and antimicrobial activity represent a promising biomaterial for bone defect reconstruction.

**Supplementary Information:**

The online version contains supplementary material available at 10.1186/s13036-021-00273-6.

## Introduction

Bone defect requires surgical repair to regenerate its function. The defect site requires scaffold to induce migration, ingrowth and osteogenic differentiation of surrounding cells. Scaffold materials must have good mechanical properties and cellular compatibility, orderly porous structure and osteoinduction capability. The most commonly used materials for the repair of bone defect include allografts, xenografts, metals [1], inorganic materials [2] and polymer materials [3].

Allografts and xenografts are the most commonly used bone repair materials for bone repair in early clinical practice [4]. Although the immune rejection of allografts is low, there are still some shortcomings, such as the limited source of grafts and the risk of infection in the donor part [5]. Although the source of xenograft bone is extensive, it also has the risk of immune rejection [6]. Both methods will be phased out with the development and application of new synthetic materials.

Synthetic polymer materials have made great progress in the field of bone defect repair due to its advantages of extensive artificial synthesis, good mechanical properties, the use of biodegradable polymers and great potential for surface modification. Dinghua Liu et al. used three-dimensional (3D) printing technology to prepare a polycarbonate (PCL) porous scaffold containing strontium-containing hydroxyapatite (HA), which effectively promoted osteogenic differentiation and bone defect repair [7]. Polymer materials generally lack biological activity. Thus, they need to be combined with other biologically active materials or surface-modified to meet the requirements of bone defect repair. Lei Chen et al. bound BMP-2 and ponericin G1 onto the surface of PLGA scaffolds via poly(dopamine). This kind of surface modification of PLGA scaffolds enhanced its osteoinduction and antimicrobial activity [8]. Xiaoyuan Li et al. bound gelatin/nanohydroxyapatite and antimicrobial peptide onto the surface of PLA scaffolds, and this surface modification method enhanced the cellular adhesion, calcium deposition and antimicrobial ability of PLA scaffolds [9].

Inorganic materials, such as HA, β-tricalcium phosphate and calcium–phosphate cement, have been widely used in bone defect repair scaffolds due to their biocompatibility and osteoconductive features [2, 10]. However, the osteogenesis capability of these materials is often insufficient and several additives (such as growth factors [11], trace elements [12, 13], stem cells [14], etc.) need to be added to accelerate osteogenic induction. Bone morphogenetic protein 2 (BMP2) has been widely used in bone repair scaffolds as a growth factor that can promote osteogenesis. The combination of BMP2 and biomaterials mainly includes blending [11, 15, 16], covalent bonding [17, 18] and specific binding [19]. Daixu Wei et al. used Soybean lecithin (SL) to encapsulate BMP2 and then prepared microspheres and scaffolds using the oil-in-water emulsion-solvent evaporative method and modified solid–liquid phase separation method. These microspheres and scaffolds showed strong abilities of stem cell attachment and proliferation, matrix mineralization abilities [15, 16]. Bing Chen et al. used genetic engineering recombination technology to introduce von Willebrand polypeptide into BMP2; this polypeptide can bind BMP2 onto the surface of collagen material and improve the utilisation rate of BMP2, thus accelerating the osteogenesis capability of scaffold [19]. Various kinds of growth factors have been applied with special binding technology to enhance their binding capability on the material surface and obtain good tissue repair effect. Ito, Yoshihiro et al. introduced 3,4-dihydroxyphenylalanine (DOPA) molecule at the end of insulin-like growth factor 1 (IGF1); the adhesion efficiency of DOPA-IGF1 on the Ti surface was enhanced due to the adhesion capability of DOPA [20]. To find a specific binding peptide with HA, Matthew L. Becker et al. used phage display technology to identify a peptide sequence that exhibits high specificity binding to HA [21]. The sequence of this polypeptide is SVSVGMKPSPRP and was named HABP. The above studies have proven that when growth factors are modified with binding peptides, their stability on the material surface can be significantly enhanced, thus enhancing their own biological function.

In this study, we used HA binding domain (HABD) to enhance the binding capability of BMP2 mimetic peptide (BMP2-MP) and PSI10 with HA to increase the osteoinduction and antibacterial capability of 3D-printed HA scaffolds. BMP2-MP (KIPKACCVPTELSAISMLYL) can promote osteogenesis-related gene expression, production of extracellular matrix and osteogenetic differentiation [22]. An antimicrobial peptide PSI10 (RRWPWWPWRR) was designed according to the research shows strong bactericidal activity. The minor inhibitory concentration (MIC) of PSI10 against to Escherichia coli (E.coli), Salmonella typhimurium, Bacillus subtilis, Staphylococcus aureus are 2, 4, 2 and 2 µg/ml, respectively. PSI10 also had a good antibacterial effect on methicillin-resistant Staphylococcus aureus (MRSA) and vancomycin-resistant Enterococcus faecium (VRE) [23]. Given the advantages of the cooperative effect of HABD with BMP2-MP and PSI10, HABD can enhance the osteoinduction and antibacterial capability of 3D-printed HA bone defect scaffolds. The osteoinduction and antibacterial activity of HA scaffolds had been intensively studied and evaluated *in vitro*.

## Materials and methods

Commercially pure HA (average diameter < 200 nm) were purchased from MACKLIN Co., Ltd. BMP2-MP, BMP2-MP/HABP, PSI10, PSI10/HABP and their corresponding fluorescein isothiocyanate (FITC)-labelled polypeptides were synthesised by Sangon Biotech Co., Ltd. Pluronic F-127 was purchased from Sigma.

### Manufacture of 3D-printed bone defect scaffolds

The geometry of the scaffold (Ф8mm×H3mm) was designed by 3DMAX 2017 software and exported in STL format. Then, the STL file was imported into Simplify3D software for print parameter setting. The filling rate was set at 50 %, the printing speed was set at 6 mm/s and the filament angles from layer to layer were set at 0°, 45°, 90° and 135°. The printing ink was a mixture of HA and Pluronic F-127 aqueous solution (20wt%) at a 50 %:50 % (W/V) ratio. The printing ink was stirred vigorously for 15 min and then filled into a 2.5 ml syringe. The inner diameter of the printer head was 0.4 mm. The biological 3D printer (3D-BioART, UB biotech. Co., Ltd) was used for scaffold printing. Finally, all scaffolds were dried overnight and sintered at 1100 °C for 3 h using a muffle furnace (Blue M LGOTM, Thermo).

### Characterisation of 3D-printed scaffolds

Micro-computed tomography (micro-CT) (SKYSCAN 1275) was used to scan and reconstruct the scaffold. Then, the porosity and pore properties of the scaffold were calculated and observed based on the reconstruction data. The microstructures and surface topography of the scaffolds were observed by optical microscopy (Olympus IX71) and scanning electron microscopy (SEM, Merlin Compact; Zeiss, Germany) in 100×, 1 K× and 10 K× magnification. The phase compositions of HA scaffolds were analysed by X-ray diffraction (XRD). The compression strength of the HA scaffold before and after sintering was tested by universal mechanics testing machine (Instron 3400).

### Fabrication of BMP2-MP/HABP and PSI10/HABP-modified scaffolds

BMP2-MP/HABP (500 ng/mL), BMP2-MP (500 ng/mL), PSI10/HABP (100 µg/mL) and PSI10 (100 µg/mL) solutions were dissolved in phosphate-buffered saline (PBS). The HA scaffolds were immersed in 1ml different solutions for up to 12 h at 4 °C with shaking (60 rpm) to enable the homogeneous dispersion of polypeptides. These surface-modified scaffolds were rinsed thrice with PBS to remove unbound polypeptides. SEM was used to evaluate the surface morphology of the scaffolds after their incubation with different polypeptides.

### Absorption and release capability of BMP2-MP/HABP and PSI10/HABP with HA scaffolds

To measure the absorption and release capability of BMP2-MP/HABP and PSI10/HABP with HA scaffolds, we incubated 1ml FITC-labelled polypeptides with the scaffolds for 12 h at 4 °C with shaking (60 rpm). Then, the scaffolds were washed thrice with PBS. All fluorescence images of scaffolds were captured by a fluorescence imaging device (CRI Maestro). The average signal data of every scaffold were calculated for statistical analysis (*n* = 3). In order to calculate the amount of polypeptide absorption and release, we measured and calculated the standard curve of polypeptide concentration and fluorescence intensity (Fig. S1). After the HA scaffold was soaked in the polypeptide solution, the fluorescence intensity of the remaining polypeptide solution was measured using a plate reader (Tecan M200), and the amount of the remaining polypeptide was calculated according to the fluorescence intensity using the standard curve. The amount of scaffold absorptive polypeptides was the total amount of polypeptides minus the amount of remaining polypeptide. To detect the polypeptide releasing ability from the HA scaffold, scaffolds were immersed in 2ml PBS solution and incubated at 4℃ with shaking (60 rpm). 100 µl of the release solution was taken at time points 1, 3, 6, 12, 24, 48 and 72 h for fluorescence detection, and the amount of polypeptide released was calculated according to the fluorescence intensity. The cumulative release rate was calculated as following formula: the cumulative release rate (%) = the amount of cumulative release of polypeptides (time point) × 100/ total amount of polypeptide absorption.

### Antibacterial activity evaluation

The inhibition zone test was used to evaluate the antimicrobial activity of different scaffolds. *Staphylococcus aureus* (*S. aureus*) and *Escherichia coli* BL21(*E. coli*) strains (bought from China Center of Industrial Culture Collection, China) were used in this test. Firstly, 100 µl bacterial solution (4.0 × 10^4^ bacteria/ml) was spread onto the LB agar plate. Then, the scaffolds were placed onto the surface of the LB agar plate. After 12 h of incubation at 37 °C, inhibition zone size was calculated by the software ImageJ.

### Cell adhesion and proliferation assays

MC-3T3-E1 cells were seeded onto different scaffolds to investigate the effect of BMP2-MP/HABP and PSI10/HABP-coated scaffolds on cellular morphology and proliferation. MC-3T3-E1 cells were purchased from the Chinese Academy of Sciences Shanghai Cell Bank and cultured in high-glucose Dulbecco’s Modified Eagle Medium (DMEM) supplemented with 10 % foetal bovine serum. The culture conditions were 37 °C in a humidified atmosphere containing 5 % CO_2_ and the medium was renewed every two days. Cell proliferation on different scaffolds were determined using the Cell Counting Kit-8 (CCK-8, Dojindo, Japan) assay. Firstly, the scaffolds were sterilised by immersion in 75 % alcohol for 30 min before coating with different polypeptides. The polypeptides were sterilised by filtration using 0.22 μm filter membrane (Millipore). Then, MC-3T3-E1 cells were seeded on different scaffolds at the density of (5 × 10^4^ cells/mL). After 3 days of culture, the medium was replaced by CCK-8. After 3 h of incubation, the absorbance values at 450 nm were measured on a multifunctional microplate scanner (Tecan Infinite M200). To investigate the cell adhesion and spreading capability on the different scaffolds, we incubated the cells with 2 µM calcein AM (Beyotime) and 1 µg/ml propidium iodide (PI) for 30 min and rinsed them repeatedly with PBS. Finally, the cell/scaffold samples were observed under a fluorescence microscope (DM2500, Leica).

### Real-time quantitative polymerase chain reaction (qPCR) analysis

MC-3T3-E1 cell suspension was seeded on different scaffolds at the density of 1 × 10^5^ cells/mL and continually cultured for 7 days. The total RNA was extracted using TRIzol reagent (Sigma) following the manual. RNA concentration was measured using NanoDrop 8000 (Thermo) and diluted with RNase-free water (Solarbio) to a final concentration of 200 ng/µl. cDNA was prepared by reverse transcription of mRNA using the PrimeScript™ Kit (TAKARA). TB Green® Fast *q*PCR Mix (TAKARA) was used to evaluate the expression of osteogenic genes by *q*PCR. The reaction conditions were: 95 °C for 30 s for 1 cycle and 95 °C for 5 s to 60 °C for 10 s for 40 cycles. Gene-specific primers, including glyceraldehyde-3-phosphate dehydrogenase (GAPDH), type I collagen (COL-I), osteocalcin (OCN) and Runx2, were designed using the primer design software Beacon 5.0 (Table 1). Each gene expression value was normalised to that of the housekeeping gene, GAPDH. The results were reported as relative gene expressions. All experiments were performed in triplicate to obtain the average data.

**Table 1:** List of genes and primer nucleotide sequences

Gene annotation	Forward primer sequence	Reverse primer sequence
**RUNX2**	GCCCTCATCCTTCACTCCAAG	GGTCAGTCAGTGCCTTTCCTC
**OCN**	TCCAAGCAGGAGGGCAATAA	CAAGTCCCGGAGAGCAGCCA
**COL-1**	CTGAAATGTCCCACCAGCC	GTCCGATGTTTCCAGTCTGC
**GAPDH**	AATGTGTCCGTCGTGGATCTG	CAACCTGGTCCTCAGTGTAGC

### Western blot analysis

The results of cell culture method were consistent with those of the *q*PCR method. The total proteins in each group were extracted using radioimmunoprecipitation assay lysis buffer (Beyotime). Protein concentration was determined using the BCA™ Protein Assay Kit (PIERCE). Electrophoresis was performed using 12 % sodium dodecyl sulfate polyacrylamide gel electrophoresis with 20 µg protein per sample. The protein was transferred from the gel to the polyvinylidene fluoride (PVDF) membrane at 200 mA for 2 h. The PVDF membrane was blocked with 5 % skim milk for 2 h, immersed in a primary antibody solution and incubated at 4 °C overnight. Next, the PVDF membrane was incubated with horseradish peroxidase-labelled secondary antibody at room temperature for 2 h. The antibody dilution ratios were as follows: GAPDH: 1:2500 (ABCAM), Runx2: 1:2000 (ABCAM), COL-I: 1:2000 (ABCAM) and OCN: 1:2000 (ABCAM). The PVDF film emitted light under the reaction of an enhanced chemiluminescence substrate. X-ray film was used for exposure and photographing. The grey value of the blot was quantitatively calculated by ImageJ software [24, 25].

### Alkaline phosphatase (ALP) assay

MC-3T3-E1 cells (5 × 10^4^/well) were seeded onto scaffolds and cultured in high-glucose DMEM. After 7 days, the ALP activity of one group was assayed using a 5-bromo-4-chloro-3-indolyl phosphate/nitro blue tetrazolium ALP colour development kit (Beyotime, China). ALP-positive cells were captured under a light microscope (Olympus IX71) in five randomly selected fields. Data are presented as mean ± standard deviation (SD), with *n* = 3. A cell lysate kit (Beyotime, China) was used in another group of cells to extract the total proteins in accordance with the specifications. ALP activity was assayed with a p-Nitrophenyl Phosphate (pNPP) Liquid Substrate System (Sigma) following the manufacturer’s instructions. A total of 200 µl pNPP solution was added to each well containing 10 µl cell lysate. The plates were incubated at room temperature for 30 min and activities were detected and read at a wavelength of 405 nm (Thermo MK3). The protein concentration of cell lysates was quantified using the BCA Kit (Beyotime, China). ALP enzyme activity was calibrated using protein concentration.

### Statistical analysis

All results were expressed as means ± SD. One-way analysis of variance with post hoc test was used to determine significant differences. *p* < 0.05 was considered statistically significant difference and the data were indicated with (*) for probability less than 0.05 (*p* < 0.05).

## Results and discussion

### Scaffold design and printing

**Fig. 1:**
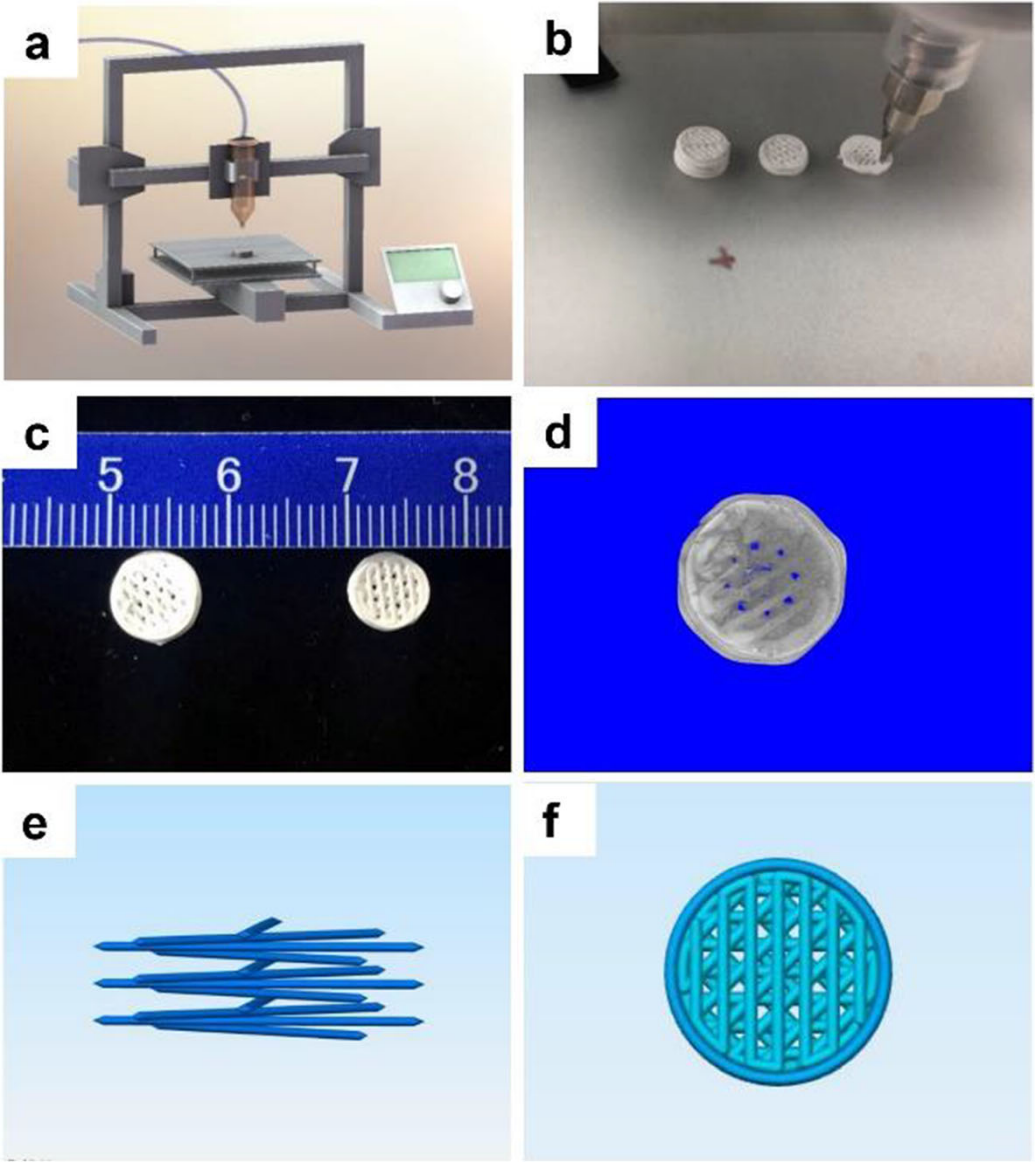
The preparation process and macroscopic photos of 3D-printed HA scaffolds. **a** Photograph of the 3D printer. **b** HA scaffold printing process. **c** comparison of HA scaffold before (left) and after (right) sintering. **d** the micro-CT images of HA scaffold before (left) and after (right) sintering. **e** filament orientation and **f** scaffold design image

The 3D-printed HA scaffolds were prepared by a biological 3D printer (Fig. 1a). Images of each part of 3D printer see Fig. S2. The HA printing ink was extruded by 0.15 MPa nitrogen to print out a computer-designed HA scaffold (Fig. 1b). The scaffold diameter decreased from 8 mm before sintering to 6 mm afterward mainly because F127 in the printing ink was burned up, causing the scaffold to shrink (Fig. 1c and d). The micro-CT scan showed a clear image of the HA scaffold, regular pore arrangement and connection. The porosity of HA scaffold before and after sintering was 27.45 and 26.53 %, respectively (Fig. 1d).

In this study, the filament angle between the scaffold layers was set to 45° (Fig. 1e). Given this fibre orientation pattern, the scaffold was viewed from top to bottom and the pore pass-through area accounted for a small proportion of the area (Fig. 1f). This filling style was selected to allow the cells to thoroughly fill the interior of the scaffold and prevent them from rapidly flowing out of the pore. In many previous research, the commonly used filling angles of 3D printed scaffold was generally 45° or 90°. Lei Chen et al. prepared a lithium–calcium–silicate crystal bioscaffold for osteochondral interface reconstruction. The angle between the scaffold filaments was set at 90° [26]. Wentao Dang et al. prepared a metal–organic framework nanosheet-structured scaffold for bone construction and tumour therapy. The angle between the scaffold filaments was 45° [27]. Theoretically, the cell retention ability of 45° would be better than that of 90°.

### Characterizations of HA scaffolds

**Fig. 2:**
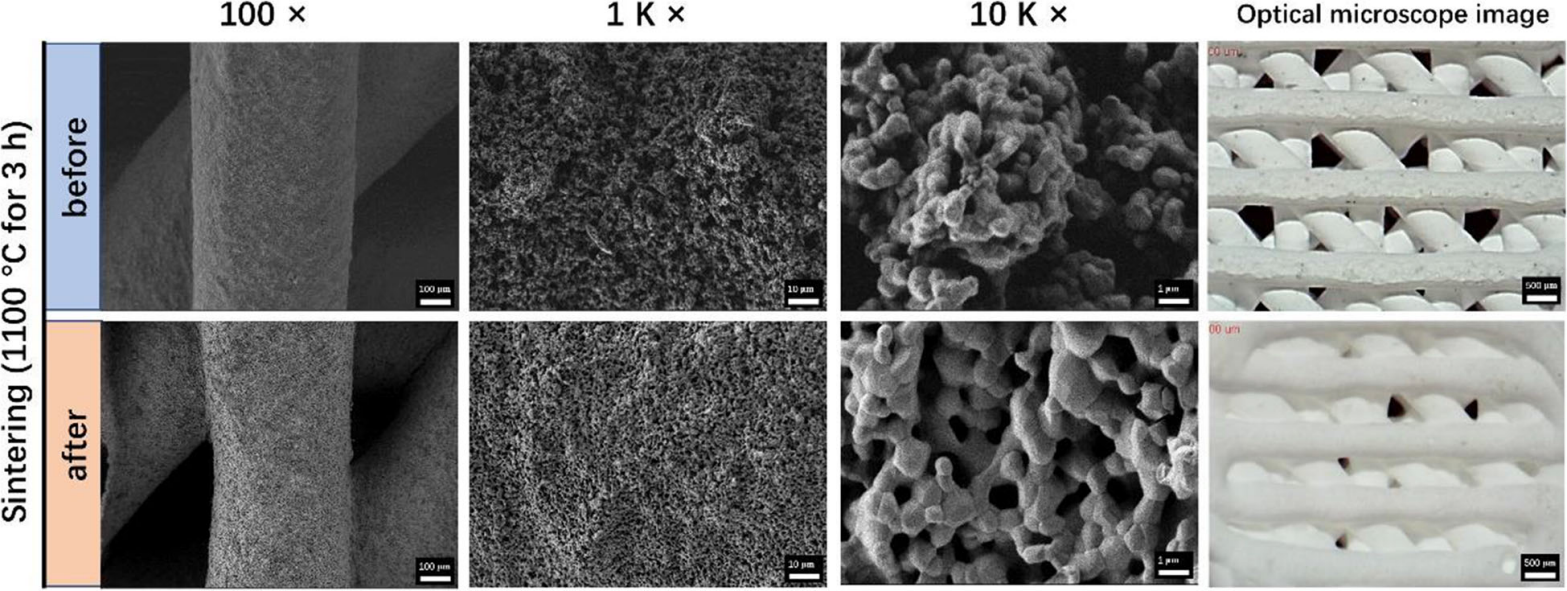
Surface morphology and XRD analysis of 3D-printed HA scaffolds (before and after sintering)

**Fig. 3:**
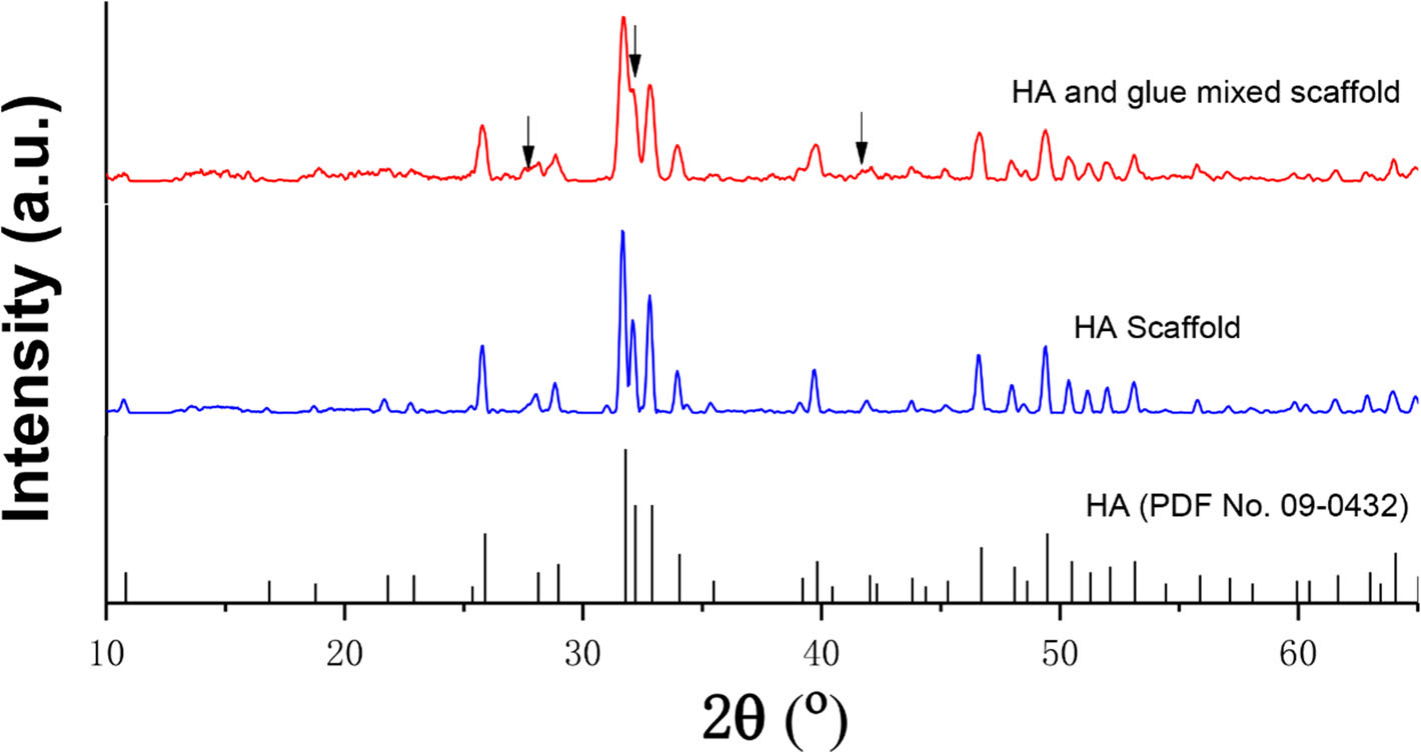
XRD patterns of HA scaffolds before and after sintering. The black arrows represent the peaks of the glue

As shown in Fig. 2, the diameter of the filament before sintering was about 490 ± 12.4 μm, whereas that after sintering was about 410 ± 16.5 μm, indicating a shrinkage of 16.4 %, which was consistent with the macroscopic size shrinkage of the HA scaffold (Fig. 1c). The SEM images showed that the diameter of HA particles was between 200 and 500 nm before sintering and the micropores were relatively dense and irregularly arranged. After sintering, the diameter of HA particles was between 500 and 1.6 μm and the micropores were relatively loose and arranged regularly. After sintering, the particles were closely connected and the crystals developed uniformly. The pore diameter of the scaffold was 584.5 ± 12.3 μm before sintering and 408.4 ± 11.4 μm after sintering, respectively. Figure 3 plots the XRD patterns of HA scaffolds before and after sintering. ‘HA and glue mixed scaffold’ and ‘HA scaffold’ represent the scaffolds without and with sintering at 1100 °C for 3 h, respectively. All patterns were compared with the means of Joint Committee on Powder Diffraction Standards. The HA scaffolds before and after sintering showed the same patterns with PDF (No. 09-0432). However, several redundant peaks appeared because of the incorporation of adhesive glue into HA powders before sintering (black arrows in Fig. 3).

The macro pore size and micro pore structure of scaffolds seriously affected the adhesion migration and osteogenic differentiation of cells around them. Tsuruga, E et al. showed that the optimal pore size for attachment, differentiation and growth of osteoblasts and vascularisation is approximately 300–400 μm [28]. Jingjing Diaod et al. prepared calcium–phosphate scaffolds with different pore sizes (100, 250 and 400 μm) and the results revealed that the scaffolds with pore structure of 100 μm were the most efficient in inducing bone formation during the repair of flat bone defects. The scaffolds with a pore size of 400 μm displayed the best capability for bone formation in the repair of long bone defects [29]. Therefore, when repairing bone defects in different positions, the pore size of the scaffold should be selected and designed in accordance with a specific condition, which will affect the repair efficiency. The pore size of the scaffold could be controlled by changing the size of the inner diameter of the printing head and the printing filling rate. The pore size of the scaffold prepared in this study is about 400 μm, so this scaffold is more suitable for repairing long bone defects.

### Absorption and release capability of BMP2-MP/HABP and PSI10/HABP with HA scaffolds

**Fig. 4:**
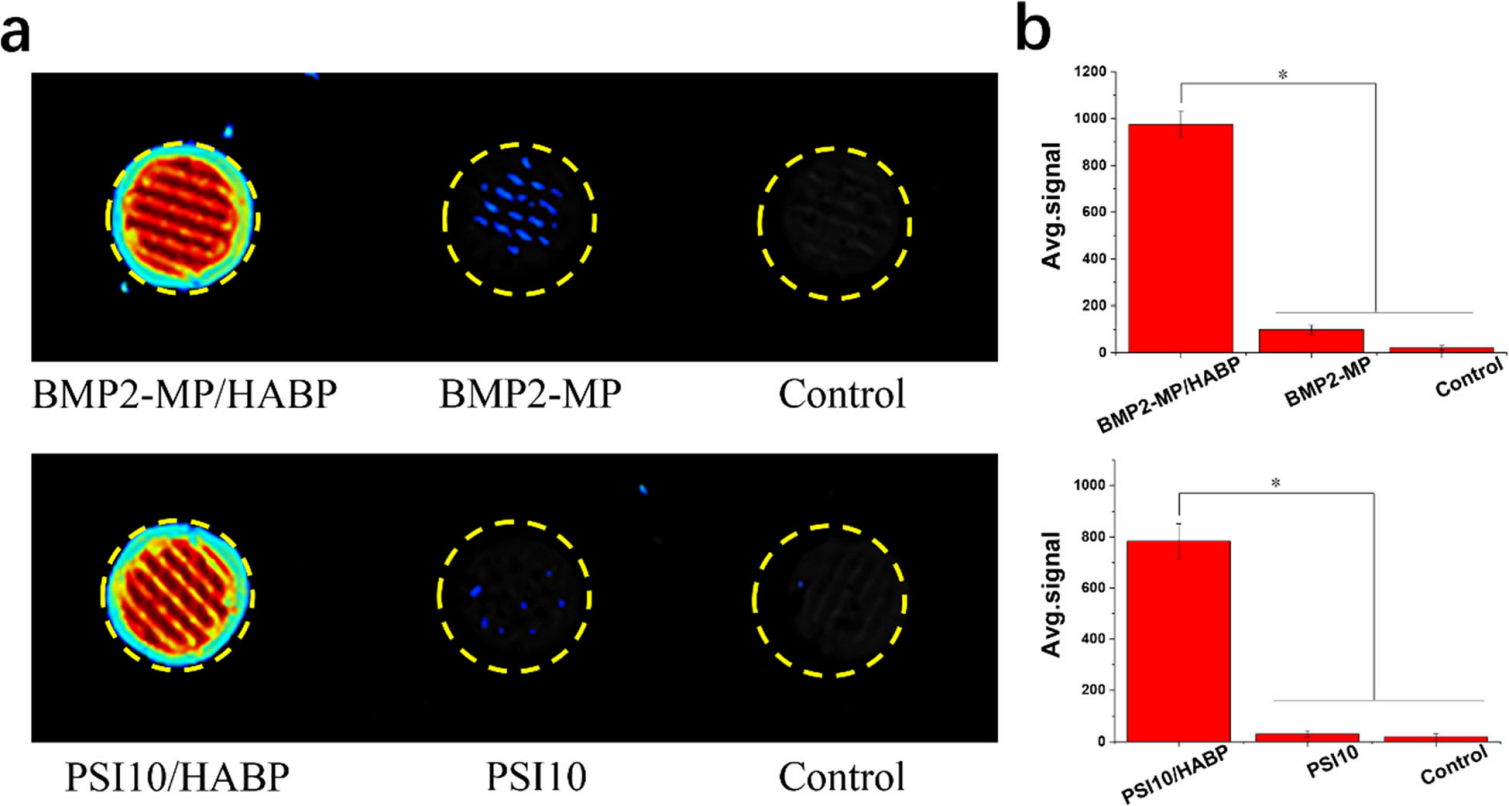
**a** Fluorescent images of HA scaffold combined with BMP2-MP/HABP, BMP2-MP, PSI10/HABP, PSI10 and control. **b** Average signal intensity on different scaffold surfaces (*n* = 3, *p < 0.05)

**Fig. 5:**
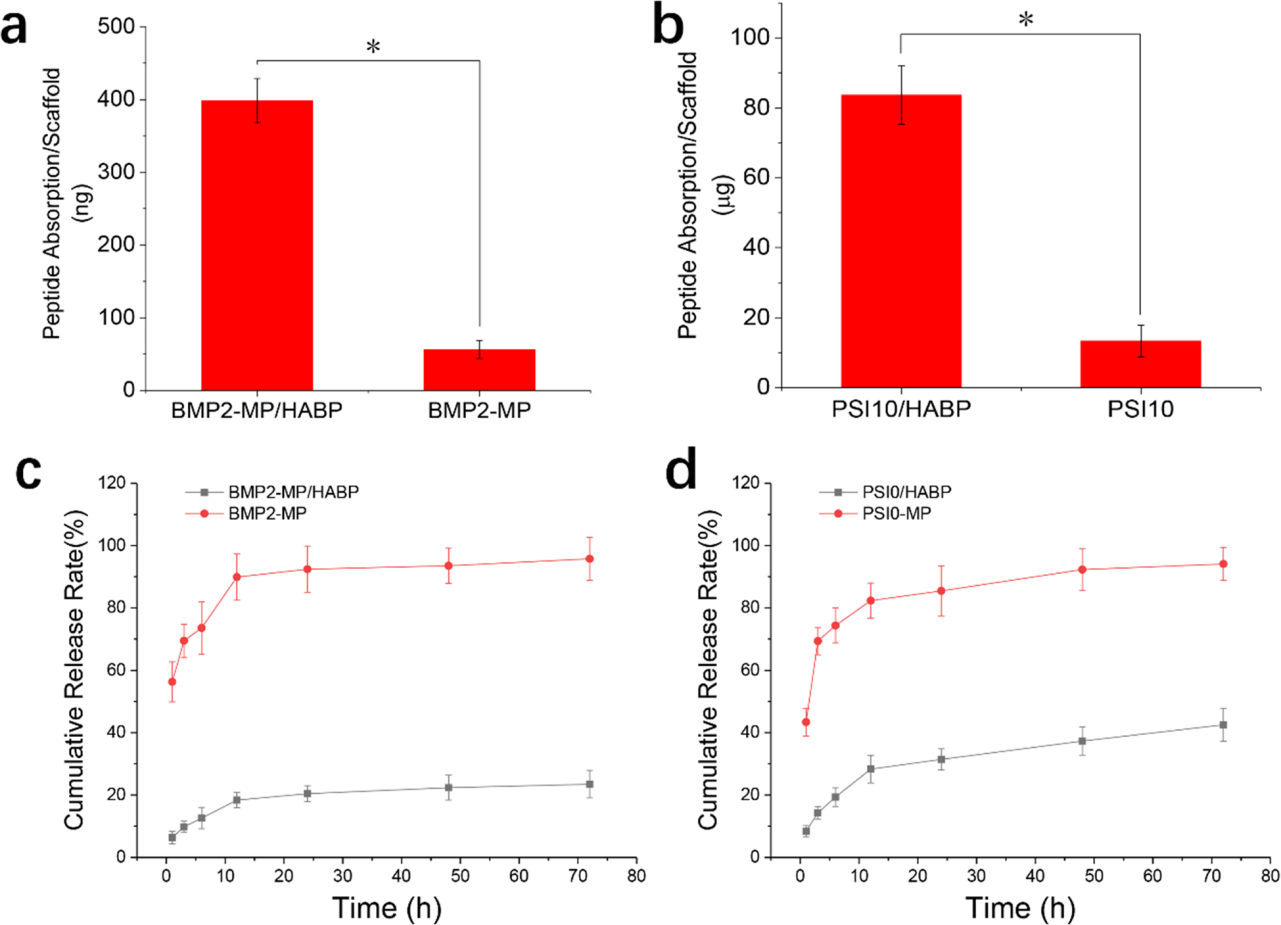
The ability of 3D-printed HA scaffolds to absorb and release polypeptides. **a** and **c** BMP2-MP and BMP2-MP/HABP, **b** and **d** PSI10 and BMP10/HABP. (*n* = 3, *p < 0.05)

**Fig. 6:**
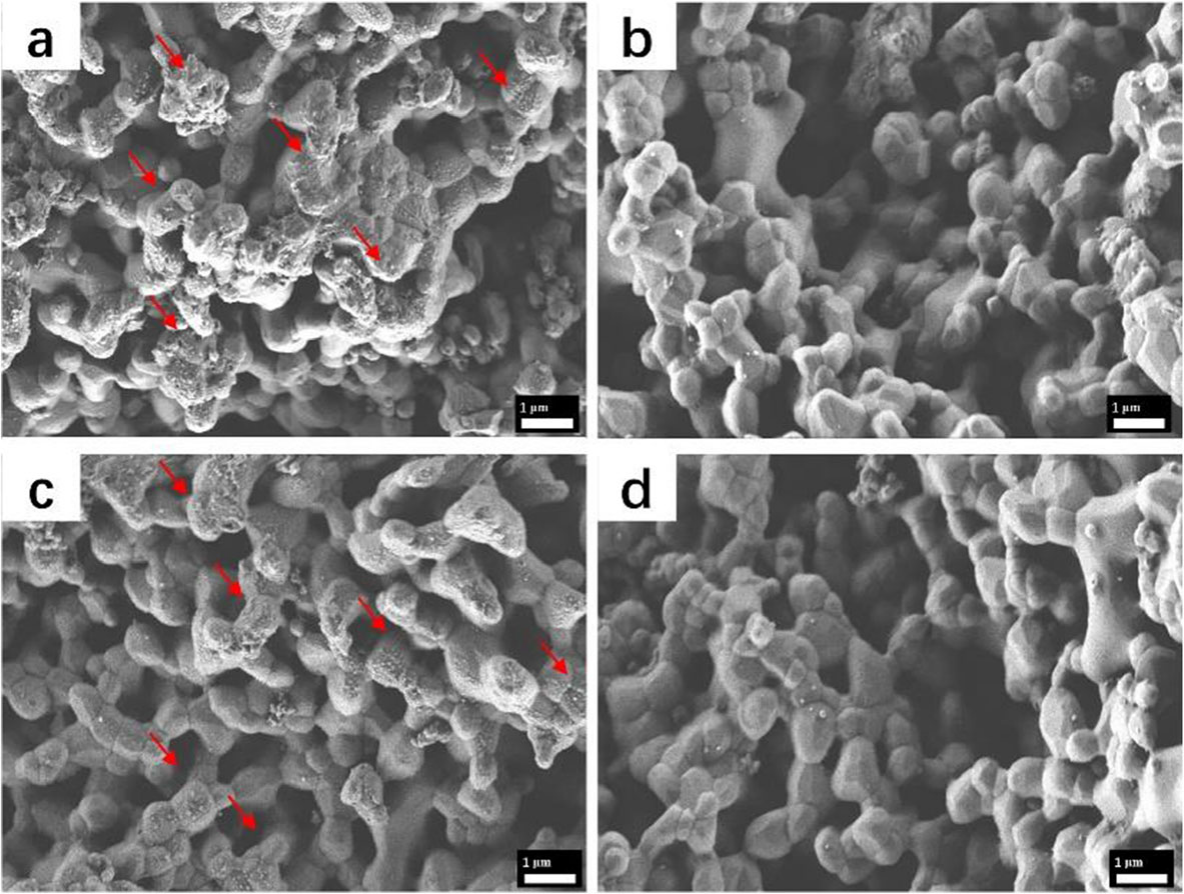
Surface morphology of HA scaffolds bound with **a** BMP2-MP /HABP, **b** BMP2-MP, **c** PSI10/HABP and **d** PSI10, the red arrow indicates the polypeptide

We used a fluorescence imager to capture coloured photos when FITC-labelled polypeptides in different groups were incubated with HA scaffolds (Fig. 4a). The heat map of the image represents the amount of polypeptide on the scaffolds. The redder the picture, the more polypeptides that were present. When the HABP tag was added to the BMP2-MP and PSI10 terminals, the binding capability to the HA scaffold was significantly enhanced compared with the no-tag polypeptides. As shown in Fig. 4b, the average signal intensities of BMP2-MP/HABP and PSI10/HABP were 9.91 and 11.16 times higher than that of BMP2-MP and PSI10, respectively. The amount of BMP2-MP/HABP and BMP2-MP absorbed by the HA scaffolds were 398.41ng and 56.31ng, respectively (Fig. 5a). The amount of PSI10/HABP and PSI10 absorbed by the HA scaffolds were 83.67 and 13.34 µg, respectively (Fig. 5b). As shown in Fig. 5c and d, the polypeptides with HABP joint were released slowly, and the cumulative release rates of BMP2-MP/HABP and PSI10/HABP were 23.45 and 42.54 % during the release period of 72 h. Interestingly, the cumulative release rates of BMP2-MP and PSI10 were 95.76 and 94.11 %, respectively. The above results indicate that the HABP tag in the polypeptide can specifically bind with the HA material to enhance the loading capacity and slow release capability. SEM was used to observe and evaluate the changes in the surface morphology of HA scaffolds bound with different groups of peptides. As shown in Fig. 6, in the BMP2/HABP and PSI10/HABP groups, HA particles exhibited a rough surface and a large number of uniform polypeptides were deposited (size range: 10–30 nm). In the BMP2-MP and PSI10 groups, the surface of HA particles was relatively smooth and showed a small amount of polypeptide deposition. The above results also prove that the introduction of HABP tag enhanced the binding capability of polypeptide to the HA scaffold and the roughness of scaffold surface. In addition, as shown in Fig. S3, EDS experiments were used to scan the elements on the surface of different scaffolds, and the results showed that the scaffolds contained the HABP polypeptide displayed higher nitrogen content than other groups, which further proved the binding ability of HABP and HA.

The HABP polypeptide contains 12 amino acids that belong to a kind of solid-binding peptides (SBPs). SBPs are polypeptides with 7–12 amino acids. They are characterised by their capability to identify a certain kind of material and bind to its surface. SBPs act similar to a ‘bridge’ between biological active factors (peptides and growth factors) and materials (metals, inorganic materials and carbohydrates), endowing materials with effective biological functions. The most important function of SBPs is the increase in binding force between bioactive molecules and materials and realisation of their slow release [30].

### Antibacterial

**Fig. 7:**
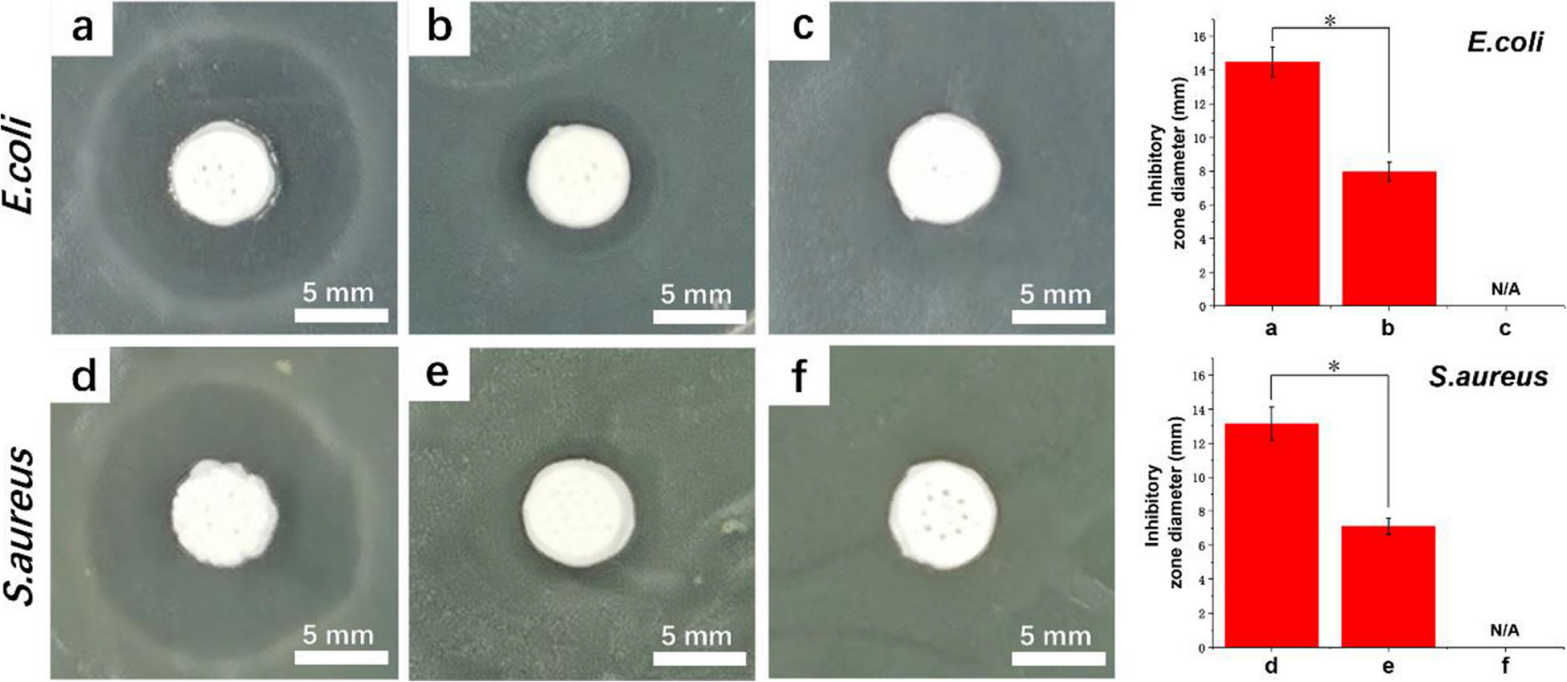
Photographs of bacterial inhibition zone and diameter changes in inhibition zones of HA scaffolds bound with PSI10/HABP (**a** and **d**), PSI10 (**b** and **e**) and control (**c** and **f**), *n* = 3, *p < 0.05

*E. coli* and *S. aureus* strains were used as model strains to evaluate the antibacterial capacity of scaffolds by calculating and observing the diameter of the antibacterial ring. Because *E. coli* belongs to the Gram-negative strain and *S. aureus* belongs to the Gram-positive strain, they are commonly used to evaluate antimicrobial capacity. As shown in Fig. 7a, when the HABP tag was introduced into the PSI10 polypeptide terminal, the scaffold showed a significant antibacterial effect compared with the PSI10 group with *E. coli* and *S. aureus.* The bacterial inhibition zones of PSI10/HABP@HA and PSI10@HA scaffolds on *E. coli* plate were 14.47 ± 0.89 and 7.97 ± 0.58 mm, respectively. When the HABP tag was introduced, the bacterial inhibition zone of the scaffold bound to the HABP/PSI10 polypeptide was 1.82 times that of PSI10 polypeptide. In the *S. aureus* group, the bacterial inhibition zones of HABP/PSI10 and PSI10 scaffolds were 13.15 ± 0.97 and 7.12 ± 0.48 mm, respectively. The bacterial inhibition zone of HABP/PSI10@HA scaffold was 1.85 times that of PSI10 (Fig. 7b). Further experiments were carried out to demonstrate the antibacterial activity of the scaffolds by live and dead staining, see Fig. S4. The experimental results were consistent with the results of bacterial inhibition zone. The enhancement of antibacterial capability was mainly due to the specific binding capability of HABP and HA materials, which led to the firm binding of PSI10/HABP antimicrobial peptides on the surface of HA particles (Fig. 6).

An excellent bone defect repair scaffold should not only have good osteogenic induction capability but also the capability to control postoperative infection. In the preparation of bone repair scaffolds, the addition of antibacterial molecules is an effective method to prevent postoperative infection [31, 32]. Huan Sun et al. used calcium–phosphate and berberine to prepare 3D printing bio-inks and printed a bone repair scaffold with antibacterial capability. Berberine played an antibacterial role and as a kind of traditional chinese medicine, it can effectively inhibit gram-negative and gram-positive bacteria. In order to ensure the antibacterial activity of the scaffold, which had not been sintered; thus, the mechanical properties of the scaffold were poor and the porosity was low [33]. As shown in Fig. S5, the compression strength of HA scaffolds before and after sintering were 20.67 ± 1.10 MPa and 26.83 ± 1.40 MPa, respectively. It can be seen from the above results that the compression strength of the scaffold increased by 29.8 % after sintering. The scaffolds prepared in our study were sintered to obtain HA scaffolds with a suitable pore structure. Then, the antibacterial peptide PSI10 with HA-specific binding capability was used to bind to the scaffold surface to achieve a long-term antibacterial effect. This antibacterial strategy is suitable for inorganic 3D-printed bone repair scaffolds.

### Cytocompatibility of HA scaffold binding with PSI10/HABP and BMP2-MP/HABP

**Fig. 8:**
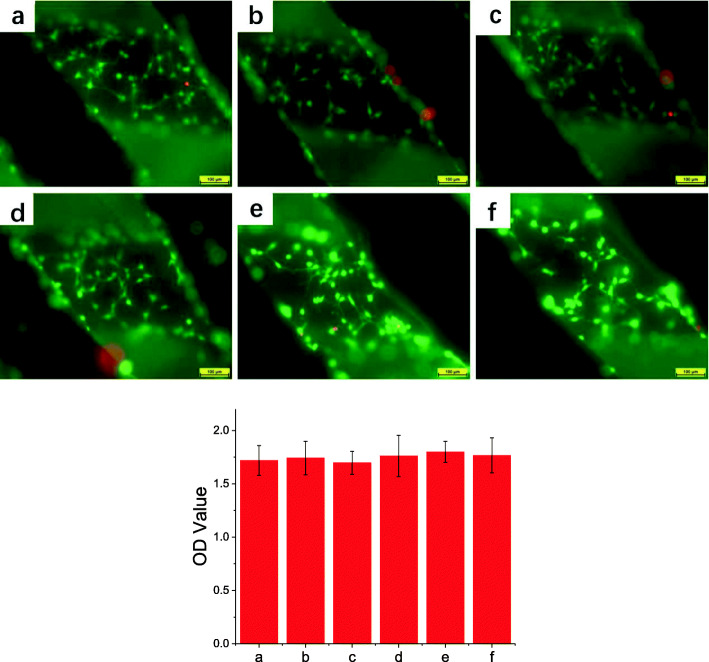
Images of live and dead staining and CCK-8 assay of Mc-3T3-E1 cells cultured on **a** HA, **b** PSI10@HA, **c** PSI10/HABP@HA, **d** BMP2-MP@HA, **e** BMP2-MP/HABP@HA and **f** PSI10/HABP&BMP2-MP/HABP@HA scaffolds for 3 days. Bar = 100 μm

**Fig. 9:**
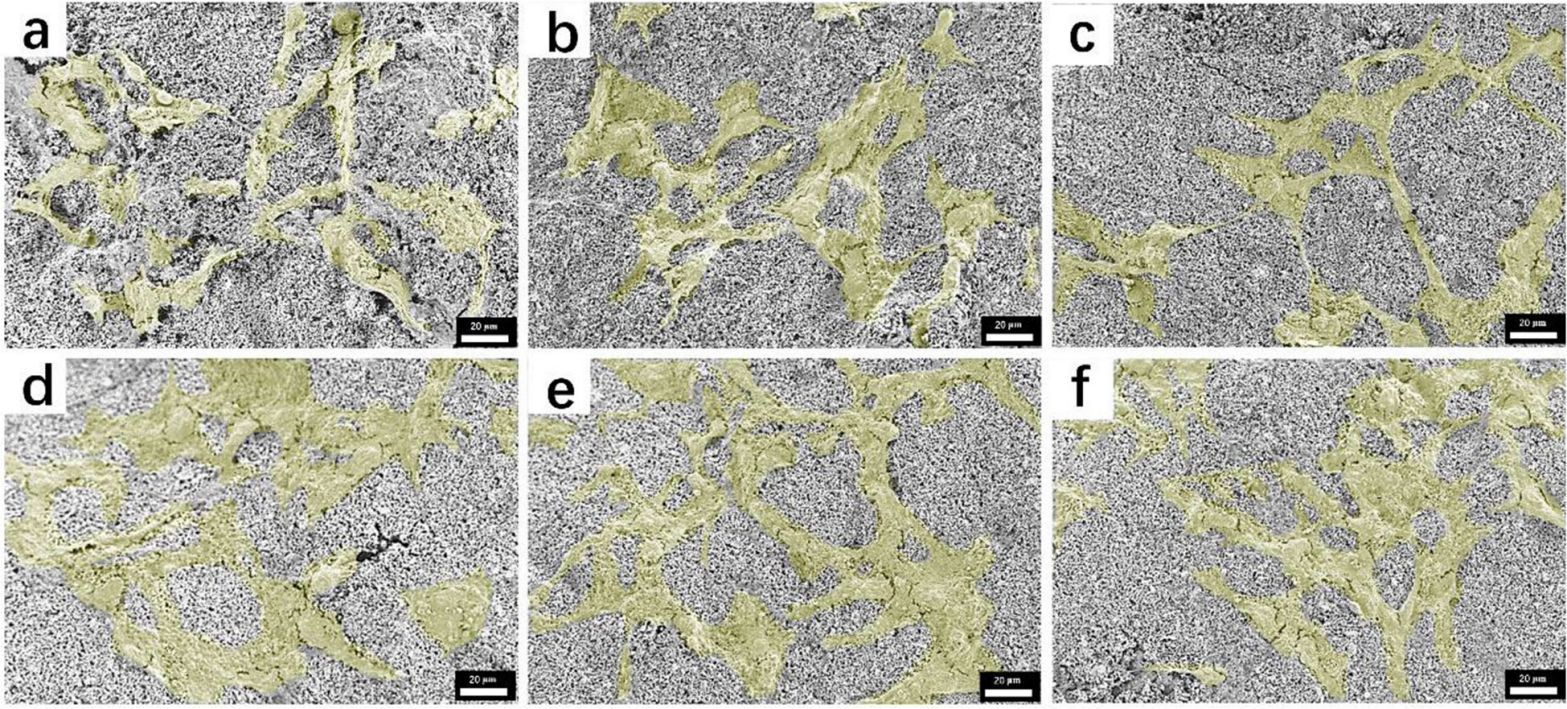
SEM images of MC-3T3-E1 cells cultured for 3 days on different scaffolds (**a** HA, **b** PSI10@HA, **c** PSI10/HABP@HA, **d** BMP2-MP@HA, **e** BMP2-MP/HABP@HA and **f** PSI10/HABP&BMP2-MP/HABP@HA). (The yellow areas represent areas of cell adhesion, scale bar = 20 μm)

To evaluate the cytocompatibility of 3D-printed HA scaffolds combined with PSI10/HABP and BMP2-MP/HABP, we stained Mc-3T3-E1 cells grown on different groups of scaffolds with calcein-AM and PI. In Fig. 8, the green fluorescent image indicates living cells growing on the scaffolds. The results show that all the scaffolds achieved good cell adhesion capability, a large number of cells grew on the scaffold surface and the morphology was intact. After the cells were stained with PI, the dead cells were almost invisible on the scaffold. The introduction of the HABP tag into the PSI10 and BMP2-MP terminals enhanced their capability to bind to the HA scaffold without affecting cell adhesion. CCK-8 results showed no significant difference in cell proliferation among all groups, which was basically consistent with the results of cell adhesion. We used SEM to acquire images of growing cells on the scaffold to further observe their morphology. As shown in Fig. 9, a large number of cells grew on the surface of each scaffold and the extracellular matrix secreted by the cells tightly wrapped the scaffold filaments. The above results indicate that after surface modification, HA scaffolds would not affect cell adhesion and proliferation.

HA is an excellent bone repair material and similar to bioapatite in natural bone in terms of composition and crystallography [34]. HA dissolves slightly in the body, releases calcium ions, participates in body metabolism, has a stimulating or inducing effect on bone hyperplasia, promotes the repair of defect tissue and shows biological activity. Our cell adhesion results reveal the excellent cell adhesion capability of the 3D-printed HA scaffold. A complete and full cell morphology was observed on the HA scaffold, whereas the scaffold surface was covered with considerable amount of extracellular matrix. After the scaffolds were coated with PSI10 and BMP2-MP, no significant negative effects were observed on the cell adhesion of scaffolds, which indicates that the PSI10 and BMP2-MP bound on the scaffold surface were non-cytotoxic to the cells.

### Effect of BMP2-MP/HABP and BMP2-MP on the expressions of osteogenic differentiation-related marker genes and proteins

**Fig. 10:**
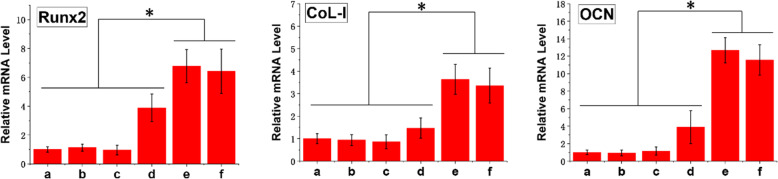
Quantitative expression of Runx2, COL-I and OCN mRNAs by *q*PCR for 3 days. **a** HA, **b** PSI10@HA, **c** PSI10/HABP@HA, **d** BMP2-MP@HA, **e** BMP2-MP/HABP@HA and **f** PSI10/HABP&BMP2-MP/HABP@HA (*n* = 3, *p < 0.05)

**Fig. 11:**
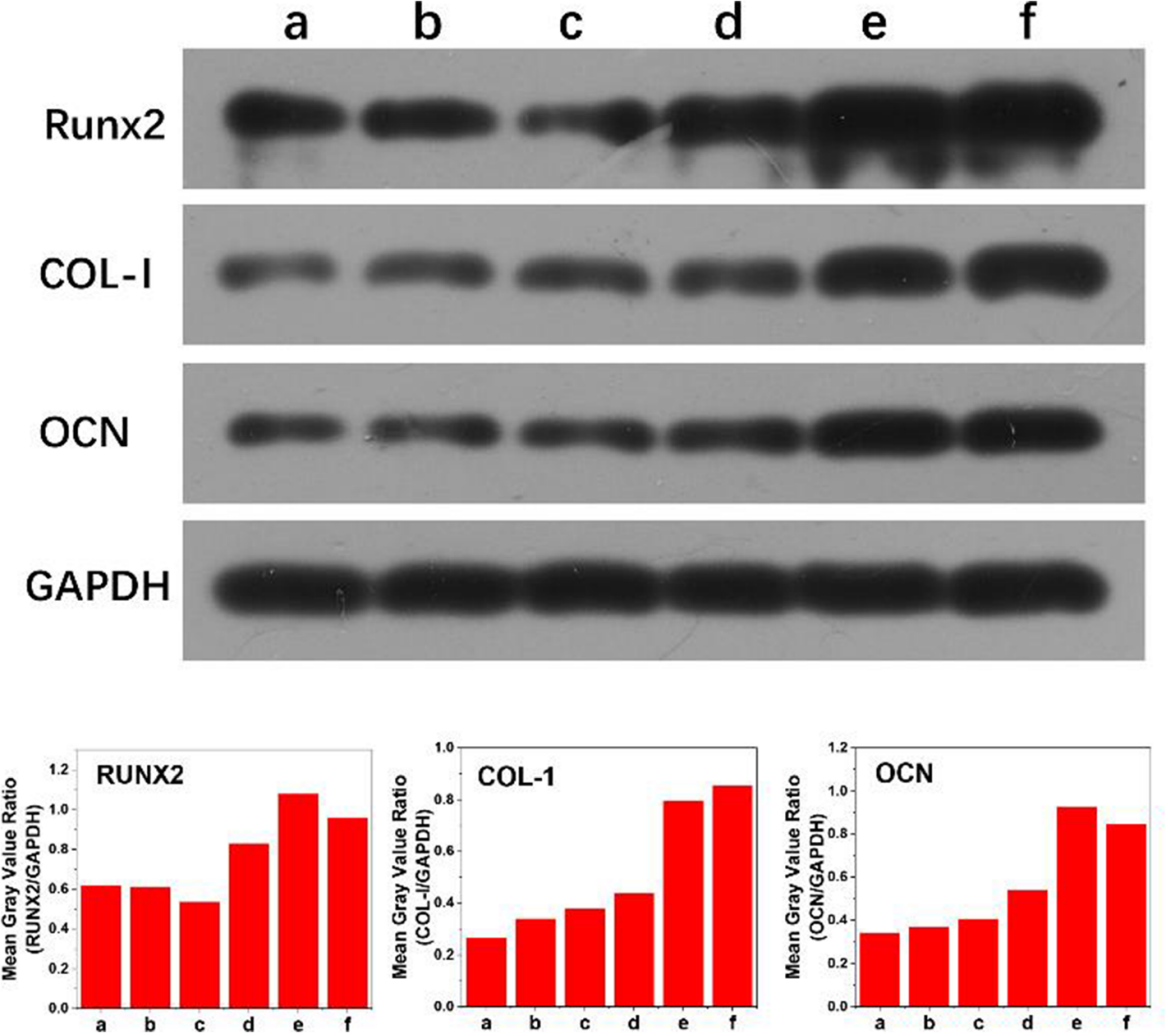
Runx2, COL-I and OCN protein expression levels were assessed by Western blot. **a** HA, **b** PSI10@HA, **c** PSI10/HABP@HA, **d** BMP2-MP@HA, **e** BMP2-MP/HABP@HA and **f** PSI10/HABP&BMP2-MP/HABP@HA scaffolds

To evaluate the effect of osteogenic differentiation of BMP2-MP/HABP and BMP2-MP, we used *q*PCR to detect the osteogenic differentiation-related marker genes and proteins (Runx2, COL-I and OCN). As shown in Fig. 10, the mRNA expression levels of Runx2, COL-I and OCN in BMP2-MP/HABP and BMP2-MP groups were significantly higher than those in HA, PSI10 and PSI10/HABP groups (*p* < 0.05). The expression levels of Runx2 in the BMP2-MP/HABP and PSI10/HABP&BMP2-MP/HABP groups were 6.78 and 6.42 times higher than those in the HA group, respectively. The expression level of COL-I in BMP2-MP/HABP and PSI10/HABP&BMP2-MP/HABP groups were 3.64 and 3.36 times that of HA group, respectively. The OCN expression levels in BMP2-MP/HABP and PSI10/HABP&BMP2-MP/HABP groups were 12.67 and 11.56 times that in the HA group, respectively. The expression levels of Runx2, COL-I and OCN in the BMP2-MP group were slightly higher than those of the HA group. The expression levels of Runx2, COL-I and OCN in the PSI10 and PSI10/HABP groups showed no significant difference from those of the HA group. Runx-2, OCN and COL-I protein expressions were also detected with Western blot on day 7 (Fig. 11). The Western blot results indicated that BMP2-MP/HABP and PSI10/HABP&BMP2-MP/HABP were more favourable of the protein expressions of Runx, COL-I and OCN than the others.

Runx2 is a transcription factor that induces immature osteocytes to differentiate into mature ones. In groups BMP2-MP/HABP and PSI10/HABP&BMP2-MP/HABP, BMP2-MP were stably bound to the surface of HA scaffolds; BMP2-MP activated the intracellular signal transduction pathways by binding to BMP receptors on the extracellular surface [22]. Firstly, the expression level of Runx2 was regulated. Subsequently, the up-regulated expression of Runx2 stimulated osteogenic differentiation of the cells via the up-regulated expression of COL-I and OCN. Western blot results also confirmed the role of BMP2-MP/HABP and PSI10/HABP&BMP2-MP/HABP in promoting osteogenic differentiation of cells. This strong osteogenic effect bound with BMP2-MP/HABP can be attributed to the contribution of HABP.

### Osteogenesis differentiation

**Fig. 12:**
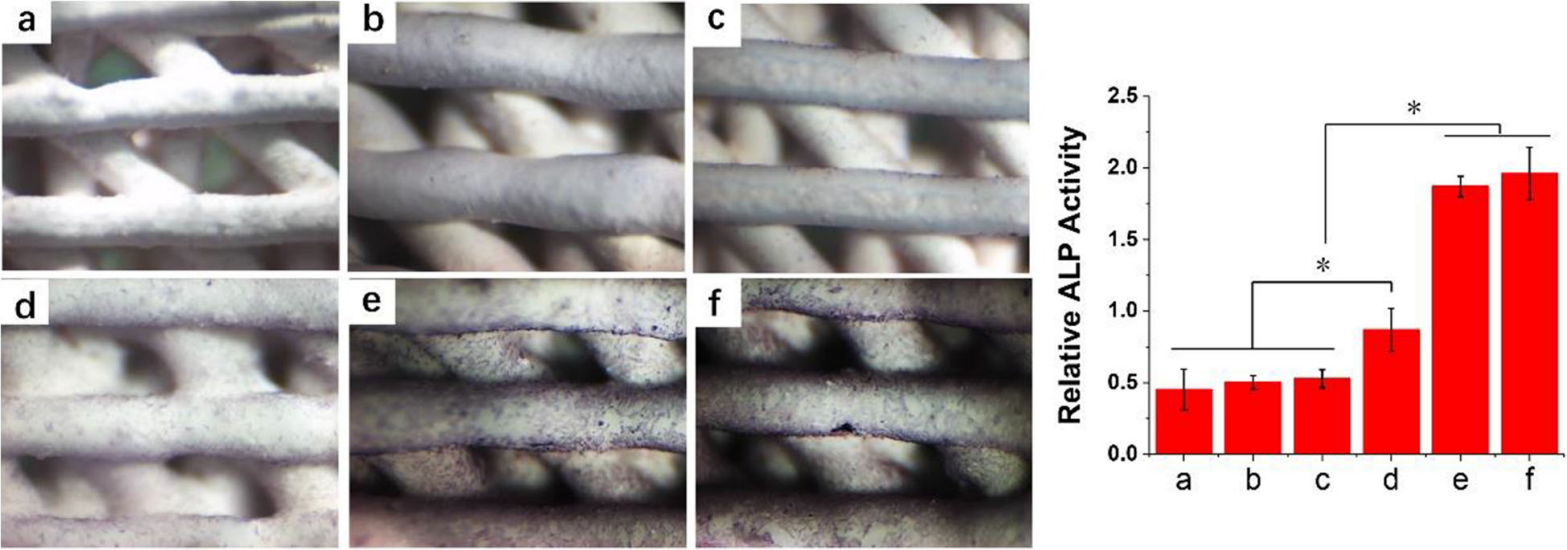
ALP staining images of MC3T3-E1 cultured for 7 days on different scaffolds. **a** HA, **b** PSI10@HA, **c** PSI10/HABP@HA, **d** BMP2-MP@HA, **e** BMP2-MP/HABP@HA and **f** PSI10/HABP&BMP2-MP/HABP@HA (*n* = 3, *p < 0.05)

The level of ALP expression is an important marker of cell osteogenic differentiation. We used ALP staining to detect and evaluate the osteoinduction capability of scaffolds bound with BMP2-MP/HABP. As shown in Fig. 12, the cells cultured on the BMP2-MP@HA group had a small number of purple-dyed cells. However, large numbers of cells were dyed purple in BMP2-MP/HABP@HA and PSI10/HABP&BMP2-MP/HABP@HA scaffolds. No cell showed evident purple staining in the HA, PSI10@HA and PSI10/HABP@HA groups. We used pNPP method to detect the ALP expression level in cells. The ALP levels of BMP2-MP/HABP@HA and PSI10/HABP&BMP2-MP/HABP@HA were 4.12 and 4.32 times that of HA, respectively.

The above results indicate that the HA scaffold combined with a large number of BMP2-MP/HABP peptides resulted in osteogenic differentiation of cells, whereas PSI10 peptides had no evident effect on osteogenic differentiation. These results were consistent with gene expression and protein levels (Figs. 10 and 11, respectively). ALP is a marker of early osteogenesis. When the activity of ALP is enhanced, it can promote the mineralisation of bone matrix. The above results also further demonstrate that when the end of BMP2-MP was added with HABP, the osteogenic effect of HA scaffold was significantly enhanced.

## Conclusions

In this study, HABP, which can specifically bind to HA materials, was introduced into the C-terminal of BMP2-MP and antimicrobial peptide PSI10. Given the strong binding capacity of HABP with HA, the HA material can stably load the BMP2-MP and PSI10. We used 3D printing technology to prepare HA scaffolds with controllable shape and porosity. When BMP2-MP/HABP and PSI10/HABP polypeptides were specifically bound to HA scaffolds, the antibacterial and osteogenic induction of scaffolds were significantly enhanced. The 3D size of bone defect can be reconstructed based on CT data and a personalised scaffold with antibacterial and osteogenic induction capability can be prepared using 3D printing technology. In future experimental study, we will evaluate the bone repair ability of these scaffolds *in vivo*, and *in vitro* evaluate the osteogenic induction ability of these scaffolds on mesenchymal stem cells and the antibacterial ability of the scaffold against other types of bacteria.

## Supplementary Information

**Additional file 1: Figure S1. **Standard curve of (a) BMP2-MP and (b) PSI10 concentration and fluorescence intensity. **Figure S2.** Images of each part of 3D printer. a print ink container, b pressure gas inlet port, c z-axis height adjustment and d print controller. **Figure S3.** EDS analysis of surface elements of different groups of scaffolds. a HA, b PSI10@HA, c PSI10/HABP@HA, d BMP2-MP@HA, e BMP2-MP/HABP@HA and f PSI10/HABP&BMP2-MP/HABP@HA scaffolds. **Figure S4.** Live and dead stains of (a)* E.coli* and (b) *S.auresu*growing on the surfaces of different groups of scaffolds. (i and iv) HA, (ii and v) PSI10@HA, (iii and vi) PSI10/HABP@HA. (i,ii and iii) stained living cells and (iv, v, and vi) stained dead cells.( Bar=25μm). **Figure S5. **Compression strength of HA scaffold before and after sintering, (*n*= 3, *p<0.05).

## Data Availability

Not applicable.

## Abbreviations

3D: Three-dimensional
HA: Hydroxyapatite
HABD: HA binding domain
BMP2: Bone morphogenetic protein 2
BMP2-MP: BMP2 mimetic peptide
PCL: Polycarbonate
DOPA: 3,4-dihydroxyphenylalanine
IGF1: Insulin-like growth factor 1
FITC: Fluorescein isothiocyanate
Micro-CT: Micro-computed tomography
SEM: Scanning electron microscopy
XRD: X-ray diffraction
*S. aureus*: *Staphylococcus aureus*
*E. coli*: *Escherichia coli* BL21
CCK-8: Cell Counting Kit-8
PI: Propidium iodide
GAPDH: Glyceraldehyde-3-phosphate dehydrogenase
COL-I: Type 1 collagen
OCN: Osteocalcin
PVDF: Polyvinylidene fluoride
SD: Standard deviation
pNPP: p-Nitrophenyl Phosphate
SBPs: Solid-binding peptides
