# Supplementary material for: Three-dimensional printed hydroxyapatite bone tissue engineering scaffold with antibacterial and osteogenic ability
Source: J Biol Eng. 2021 Aug 9;15:21. doi: 10.1186/s13036-021-00273-6 (PMC8353754; doi:10.1186/s13036-021-00273-6)
Supplement: Supplementary file 1 — Additional file 1: Figure S1. Standard curve of (a) BMP2-MP and (b) PSI10 concentration and fluorescence intensity. Figure S2. Images of each part of 3D printer. a print ink container, b pressure gas inlet port, c z-axis height adjustment and d print controller. Figure S3. EDS analysis of surface elements of different groups of scaffolds. a HA, b PSI10@HA, c PSI10/HABP@HA, d BMP2-MP@HA, e BMP2-MP/HABP@HA and f PSI10/HABP&BMP2-MP/HABP@HA scaffolds. Figure S4. Live and dead stains of (a) E.coli and (b) S.auresugrowing on the surfaces of different groups of scaffolds. (i and iv) HA, (ii and v) PSI10@HA, (iii and vi) PSI10/HABP@HA. (i,ii and iii) stained living cells and (iv, v, and vi) stained dead cells.( Bar=25μm). Figure S5. Compression strength of HA scaffold before and after sintering, (n= 3, *p<0.05). [file 13036_2021_273_MOESM1_ESM.docx]

Supplementary material

**Three-dimensional printed hydroxyapatite bone tissue engineering scaffold with antibacterial and osteogenic ability**

Liu Zhongxing ^1, †^, Wu Shaohong ^2, †^, Li Jinlong ^1*^, Zhang Limin ^3^, Wang Yuanzheng^1^, Gao Haipeng^1^, Cao Jian ^1*^

^1^ Department of Orthopedics, Affiliated Hospital of Chifeng University, Chifeng, Inner Mongolia, 024000, PR China.

^2^ Department of Stomatology, Affiliated Hospital of Chifeng University, Chifeng, Inner Mongolia, 024000, PR China.

^3^Department of Ophthalmology, Affiliated Hospital of Chifeng University, Chifeng, Inner Mongolia, 024000, PR China.

* Corresponding author: (Cao Jian) caojian2005088@sina.cn, Tel/Fax numbers +86-0476-5973358 and (Li Jinlong) cflijinlong@163.com, Tel/Fax numbers +86-0476-5973358

^†^ These authors contributed equally to this work


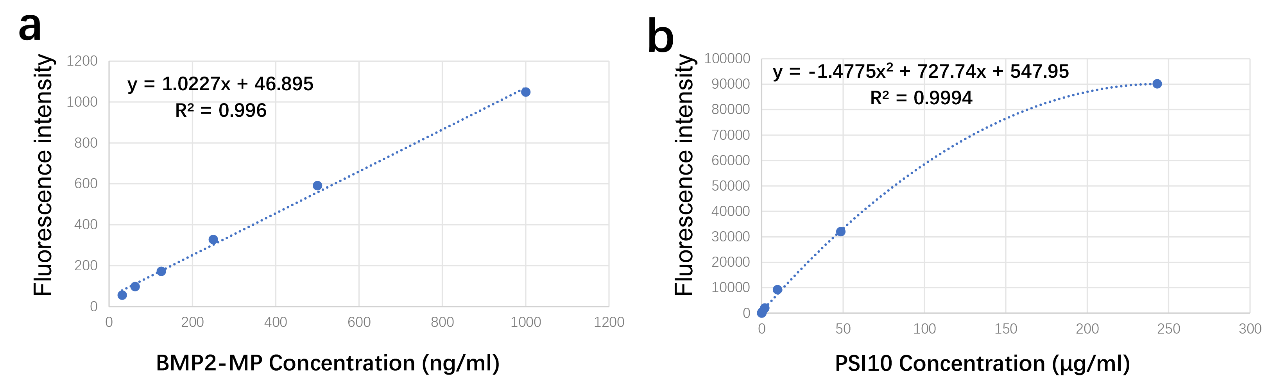


**Fig. S1** Standard curve of **(a)** BMP2-MP and **(b)** PSI10 concentration and fluorescence intensity.


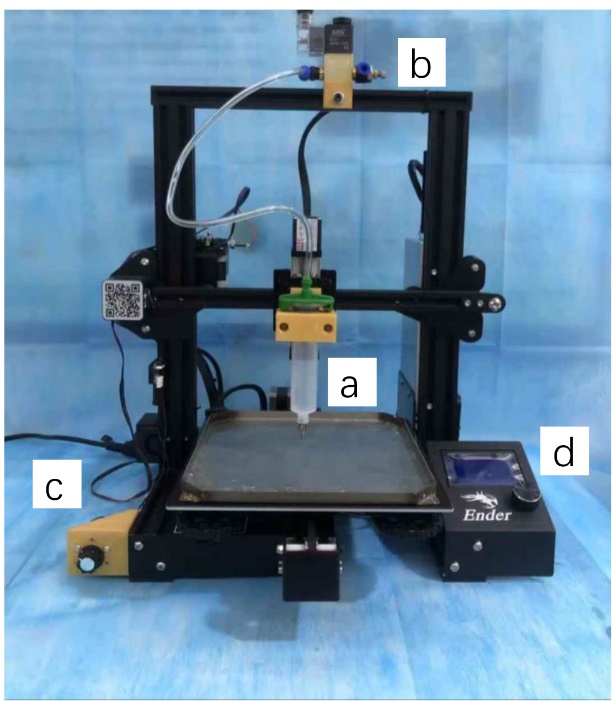


**Fig. S2**. Images of each part of 3D printer. **a** print ink container, **b** pressure gas inlet port, **c** z-axis height adjustment and **d** print controller.


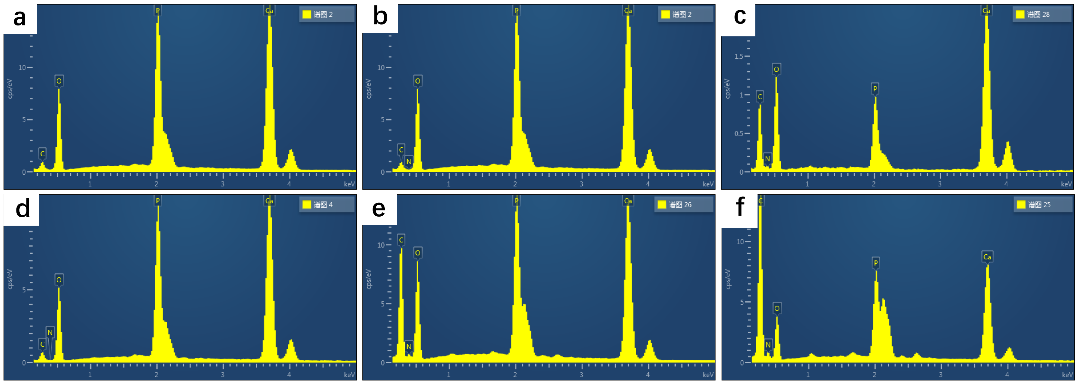


**Fig. S3**. EDS analysis of surface elements of different groups of scaffolds. **a** HA, **b** PSI10@HA, **c** PSI10/HABP@HA, **d** BMP2-MP@HA, **e** BMP2-MP/HABP@HA and **f** PSI10/HABP&BMP2-MP/HABP@HA scaffolds.


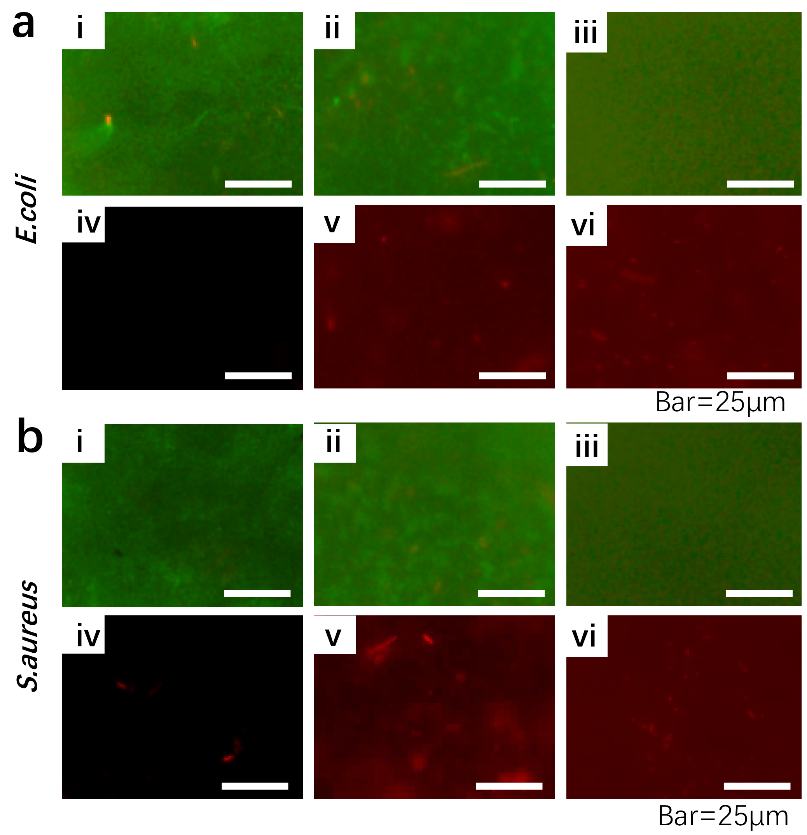


**Fig. S4.** Live and dead stains of **(a)** *E.coli* and **(b)** *S.auresu* growing on the surfaces of different groups of scaffolds. (i and iv) HA, (ii and v) PSI10@HA, (iii and vi) PSI10/HABP@HA. (i,ii and iii) stained living cells and (iv, v, and vi) stained dead cells.( Bar=25μm)

**Fig. S5** Compression strength of HA scaffold before and after sintering, (*n* = 3, **p*<0.05).
